# Retraction Note: Intracellular citrate accumulation by oxidized ATM-mediated metabolism reprogramming via PFKP and CS enhances hypoxic breast cancer cell invasion and metastasis

**DOI:** 10.1038/s41419-023-06174-4

**Published:** 2023-09-28

**Authors:** Meixi Peng, Dan Yang, Yixuan Hou, Shuiqing Liu, Maojia Zhao, Yilu Qin, Rui Chen, Yong Teng, Manran Liu

**Affiliations:** 1 Key Laboratory of Laboratory Medical Diagnostics Designed by Chinese Ministry of Education, Chongqing Medical University, Chongqing, 400016 China; 2 Experimental Teaching Center of Basic Medicine Science, Chongqing Medical University, Chongqing, 400016 China; 3 Department of Endocrine and Breast Surgery, The First Affiliated Hospital of Chongqing Medical University, Chongqing, 400016 China; 4 Georgia Cancer Center, Department of Oral Biology, Dental College of Georgia, Augusta University, Augusta, GA USA

Retraction to: *Cell Death and Disease* 10.1038/s41419-019-1475-7, published online 08 March 2019

The Editors have retracted this article because the authors have informed them that there are multiple errors in in the assembly of the images shown in Figure 8, Figure 9, Figure S1 and Figure S9. The Editors no longer have confidence in the results and conclusions presented. Meixi Peng agrees with this retraction. Dan Yang, Yixuan Hou, Shuiqing Liu, Maojia Zhao, Yilu Qin, Rui Chen, Yong Teng and Manran Liu have not responded to correspondence from the Publisher about this retraction.

